# Neck circumference as a predictor of gestational diabetes and risk of adverse outcomes in pregnancy of Brazilian woman with overweight and obesity

**DOI:** 10.20945/2359-3997000000499

**Published:** 2022-06-02

**Authors:** Camila Rodrigues de Souza Carvalho, Patricia Medici Dualib, Rosiane Mattar, Sérgio Atala Dib, Bianca de Almeida-Pititto

**Affiliations:** 1 Universidade Federal de São Paulo Programa de Pós-graduação em Endocrinologia e Metabologia São Paulo SP Brasil Programa de Pós-graduação em Endocrinologia e Metabologia, Universidade Federal de São Paulo, São Paulo, SP, Brasil; 2 Universidade Federal de São Paulo Departamento de Medicina São Paulo SP Brasil Departamento de Medicina da Universidade Federal de São Paulo, São Paulo, SP, Brasil; 3 Universidade Federal de São Paulo Departamento de Obstetrícia São Paulo SP Brasil Departamento de Obstetrícia, Universidade Federal de São Paulo, São Paulo, SP, Brasil; 4 Universidade Federal de São Paulo Departamento de Medicina Preventiva São Paulo SP Brasil Departamento de Medicina Preventiva, Universidade Federal de São Paulo, São Paulo, SP, Brasil

**Keywords:** Gestational diabetes mellitus, neck circumference

## Abstract

**Objective:**

To evaluate the association of neck circumference (NC) with gestational diabetes (GDM) and adverse outcomes in women with overweight and obesity.

**Subjects and methods:**

This prospective study included 132 (BMI > 25 kg/m^2^) pregnant women without and with GDM. Standardized questionnaire and biochemical/physical evaluation were performed during the 1^st^ to 3^rd^ trimester. Fifth-five women were evaluated regarding hypertension in pregnancy, type of delivery and neonatal complications (death, intensive care unit admission and hypoglycemia).

**Results:**

Women with (n = 61) and without (n = 71) GDM had similar mean (SD) pre-gestational BMI [30.3 (4.0) vs. 29.4 (3.5) kg/m^2^, p = 0.16]. Women with GDM were older [32 (6) vs. 28 (6) yrs, p < 0.001] and had greater NC [36.0 (2.7) vs. 34.5 (1.8) cm, p < 0.001]. NC was similar in women with GDM diagnosed in first or third trimester [p = 0.4] and was correlated with FPG [r 0.29, p = 0.01] and systolic [r 0.28, p = 0.001] and diastolic [r 0.25, p = 0.004] blood pressure. NC was associated with GDM [OR 1.25, 95%CI 1.03-1.52] adjusted for age, physical activity, education and familiar history of diabetes. In ROC analysis, the area under the curve was 0.655 and the cut-off value of 34.5 cm had 0.70 of sensitivity and 0.51 of specificity for GDM. Women who had NC ≥ 34.5 vs. < 34.5 cm had higher frequencies of hypertension [32.3 vs. 4.2%, p = 0.01].

**Conclusions:**

In a group of pregnant women with overweight or obesity, NC can be a useful tool for identifying risk of GDM and obstetric adverse outcomes.

## INTRODUCTION

Gestational diabetes mellitus (GDM) is one of the most common complications in pregnancy and affects around one in seven pregnancies or 14% of worldwide (1). The public health system in Brazil estimates an incidence of 18% of hyperglycemia in pregnancies (2). Hyperglycemia during pregnancy is a major current problem, increasing maternal and fetal risks such as pre-eclampsia, premature birth, cesarian delivery, and fetal overgrowth. In this context, it is important to identify early markers of those women most at risk of GDM and potential complications.

Among the risk factors for GDM, excess weight plays an important role, both as an important mediator of the pathophysiology of insulin resistance and for its high prevalence in populations (2), for identifying women at risk of developing GDM.

It is well established that anthropometric measures that indicate greater adiposity, such as BMI (body mass index), and, especially the centralized deposit of fat, such as waist circumference, can estimate metabolic risk (3). However, these measurements are susceptible to interpretations errors during pregnancy. In this context, neck circumference (NC) has gained the attention of health professionals, mainly because of ease in measurement, feasibility of implementation in different situations, such as during gestation, and less likelihood of being influenced by body changes that happen during pregnancy, notably in areas of normal iodine level.

NC has already been established as an indicator of the risk of metabolic syndrome, such as insulin resistance, central obesity, blood pressure and increased postprandial glucose, and triglyceride levels (4,5). It is worth mentioning that the cutoff points for the identification of individuals with the worst cardiometabolic profile vary according to the investigated population (4,5). In pregnant women, a few studies have found an association between NC and the diagnosis of GDM, with different cutoff points (4,6,7), justifying the investigation of the role of this measure in other populations.

In this context, the current study aimed to evaluate the association of NC with the diagnosis of GDM and adverse pregnancy-related outcomes in women with overweight and obesity.

## SUBJECTS AND METHODS

This is a prospective study that enrolled pregnant women over the age of 18 years and with BMI of ≥25 kg/m^2^. All pregnant women who met the inclusion criteria and received pre-natal assistance from December 2018 to August 2020 were included. A total of 143 pregnant women consented to participation, but 11 withdrew voluntarily from the study. Exclusion criteria were known autoimmune disease or chronic use of medications, particularly metformin, or inflammatory bowel disease. Pregnant women with GDM (n = 61) were recruited from the Gestational Diabetes Out-patient Clinic at the Federal University of Sao Paulo (Unifesp) and those without GDM (n = 71) from the Normal Gestation Out-patient Clinic of the Gynecologic and Obstetric Department of Unifesp. A total of 132 women were included in the study and data collection was performed. Thirty-six GDM participants and 65 non-GDM participants were enrolled since the first trimester of gestation. A total of 86 women had complete data until the post-partum period the regarding diagnosis of hypertension in pregnancy, type of delivery, and neonatal complications (death, intensive care unit admission, and hypoglycemia). During each trimester and at the post-partum period, participants were subjected to evaluation with standardized questionnaires and anthropometric data. The study protocol was approved by the Institutional review board of presentation for ethical appreciation of Unifesp, with the approval number (CAAE, 06745219.8.0000.5505).

### Standardized questionnaires

Demographic, socioeconomic and lifestyle data (physical activity assessed in minutes per week), and morbid personal and family history of the mother were obtained in the 1st and 3rd trimesters of pregnancy. If the mother is diagnosed with GDM only in the third trimester, demographic, socioeconomic, and known morbidity data were collected at this time. Education was assessed according to years at school (up to 7 -13, ≥14 years) and physically active was defined as activity for 150 min per week. Data on the time of delivery, normal or cesarian section (c-section) delivery and infant's birth weight were also collected during the assistance period.

### Anthropometry, blood pressure and medication use

Maternal weight was obtained using a digital scale with a precision of 100 g and height was measured with a precision of 0.5 cm, and BMI was calculated. NC was measured using a non-flexible tape (cm) immediately below the cricoid cartilage and perpendicular to the long axis of the neck, in seated position.

Maternal blood pressure was obtained using a mercury sphygmomanometer. Three blood pressure measurements were performed to adjust the cuff to the brachial circumference, after 5 min of rest in the seated position. The final systolic and diastolic pressure values were those that represented the arithmetic mean of the last two measurements.

The frequencies of medication use were as follows: antihypertensive drugs, 2 pregnant women with GDM; levothyroxine, 11 pregnant women, 6 GDM; fluoxetine, 2 pregnant women with GDM; aspirin, 2 pregnant women with GDM; and insulin, 29 pregnant women with GDM.

### Laboratory tests for blood collection and GDM diagnosis

Blood samples were collected after overnight fasting, and an oral glucose tolerance test (OGTT) was performed. The samples were immediately centrifuged and analyzed in a clinical laboratory. Plasma glucose level was determined with the glucose oxidase method. Total cholesterol, high-density-lipoprotein-cholesterol and triglycerides concentrations by enzymatic colorimetric methods, processed in an automatic analyzer. Low-density-lipoprotein-cholesterol and very-low-density-lipoprotein-cholesterol concentrations were obtained by difference, using the Friedewald equation. GDM diagnosis was based on the IADPSG criteria [in the 1st (FBG ≥ 92 mg/dL, n = 128) or in the 2nd or 3rd trimester (OGTT: fasting ≥ 92 and/or 1 h ≥ 180 and/or 2 h ≥ 153 mg/dL, n = 673).

### Statistical analysis

Continuous variables were presented as mean (standard deviation) and categorical variables as frequency (percentage). Clinical and laboratory variables, and maternal-fetal outcomes were compared using Student^®^ t-test (continuous variables) or chi-squared test (categorical variables) according to the diagnosis of GDM. Association of NC with continuous variables (dependent variables) during pregnancy was tested through linear regression analysis, crude and adjusted for age and pre-gestational BMI. Logistic regression analysis was performed, in which the dependent variable was GDM diagnosis and independent variable of main interest was NC during pregnancy, adjusted for the covariables of interest. NC cut off was pursued after ROC Curve analysis. Occurrence of maternal-fetal complications was compared according to neck circumference cutoff during pregnancy. The Statistical Package for the Social Sciences^®^, v 22.0 (SPSS Incorporation, 2000) was used for statistical analysis and a p value of < 0.05 was considered statistically significant.

## RESULTS

Of 132 participants, women with (n = 61) and without (n = 71) GDM had similar pre-gestational BMI mean (SD) [30.3 (4.0) *vs.* 29.4 (3.5) kg/m^2^, respectively, p = 0.164], familiar history of diabetes, physical activity, educational level, lipid profile and blood pressure measurements in pregnancy (Table 1). Women with GDM were older [32 (6) *vs.* 28 (6) years, p < 0.001] and had greater NC [36.0 (2.7) *vs.* 34.5 (1.8) cm, p < 0.001] than those without GDM.

**Table 1:** Characteristics of the women with or without gestational (GDM) diabetes

Maternal's characteristics		Without GDM (n = 71)	With GDM (n = 61)	P
Age (yrs)		28 (6)	32 (6)	<0,001
Color, White, n (%)		32 (45.1)	21 (35.0)	0.242
Family history of DM, n (%)		20 (28.2)	25 (41.7)	0.10
Education, n (%)				0.12
	Up to 7 years	1 (1.4)	5 (8.2)	
	8-13 years	57 (80.3)	42 (68.9)	
	≥14 years	13 (18,^3^)	14 (23.0)	
Physical exercise ≥150 min /week*, n (%)		30 (42.3)	26 (42.6)	0.56
Pre-gestational BMI (kg/m^2^)		29.4 (3.5)	30.3 (4.0)	0.16
Weight gain during pregnancy (kg)				
Neck circumference (cm)		34.5 (1.8)	36.0 (2.7)	<0.001
Systolic BP (mmHg)		109 (11)	115 (11)	0.005
Diastolic BP (mmHg)		68 (10)	71 (9)	0.045
TSH (mU/L)		2.4 (1.1)	2.2 (1.4)	0.415

Mean (SD); p-value, Student^®^ t test. BMI: body mass index; BP: blood pressure.

* Before pregnancy

Regarding the NC measured in different trimesters of the pregnancy, we performed some analyses comparing the measurements throughout pregnancy. Considering only NC measured in the 1st trimester, the GDM group (n = 36) had greater NC [36.0 (3.0) *vs.* 34.6 (1.8) cm, p = 0.007] than those without GDM (n = 65). Important to notice that neck circumference was similar between women with GDM diagnosed in 1st (n = 27) or third trimester (n = 34) [36.5 (2.6) *vs.* 36.0 (2.4) cm, p = 0.43], as well as pre-gestational BMI [29.8 (3.3) *vs.* 30.7 (4.4) kg/m^2^, p = 0.36].

In the crude linear regression analysis, we observed that NC was significantly associated with fasting plasma glucose and systolic and diastolic blood pressure, all variables from the gestational period. After adjustment for age and pre-gestational BMI, NC was independently associated with systolic and diastolic blood pressure and 2-h plasma glucose from OGTT, while the fasting plasma glucose turned to be borderline for this association (Table 2).

**Table 2:** Association of plasma glucose (mg-dL) and blood pressure (mmHg) with neck circumference (cm) during gestation in the study patients

	β (crude)	95% CI	p-value	β (adjusted)	95% CI	p-value
Fasting plasma glucose (mg/dL)	2.98	0.74 to 5.23	0.011	2.04	-0.95 to 5.03	0.173
1-h plasma glucose (mg/dL)	-0.42	-5.35 to 4.52	0.864	1.87	-4.75 to 8.48	0.569
2-h plasma glucose (mg/dL)	4.42	-0.66 to 9.50	0.086	7.31	0.97 to 13.66	0.025
Systolic BP (mmHg)	1.30	0.52 to 2.09	0.001	1.37	0.45 to 2.28	0.004
Diastolic BP (mmHg)	0.99	0.33 to 1.65	0.004	1.00	0.23 to 1.78	0.011

Linear regression analysis. Adjusted for age and pre-gestational body mass index (BMI). BP: blood pressure.

In the logistic regression analysis, NC was associated with GDM [odds ratio (1.25, 95% confidence interval (1.03 to 1.52) after adjustment for age, physical activity, education, and familiar history of diabetes (Table 3).

**Table 3:** Association of the diagnosis of gestational diabetes with neck circumference during pregnancy

	OR	95% CI	p-value
Neck circumference (cm)	1.25	1.03 to 1.52	0.023
Age (yrs)	1.14	1.05 to 1.23	0.002

Adjusted for physical activity, education, and familiar history of diabetes (non-significant).

**Figure 1:**
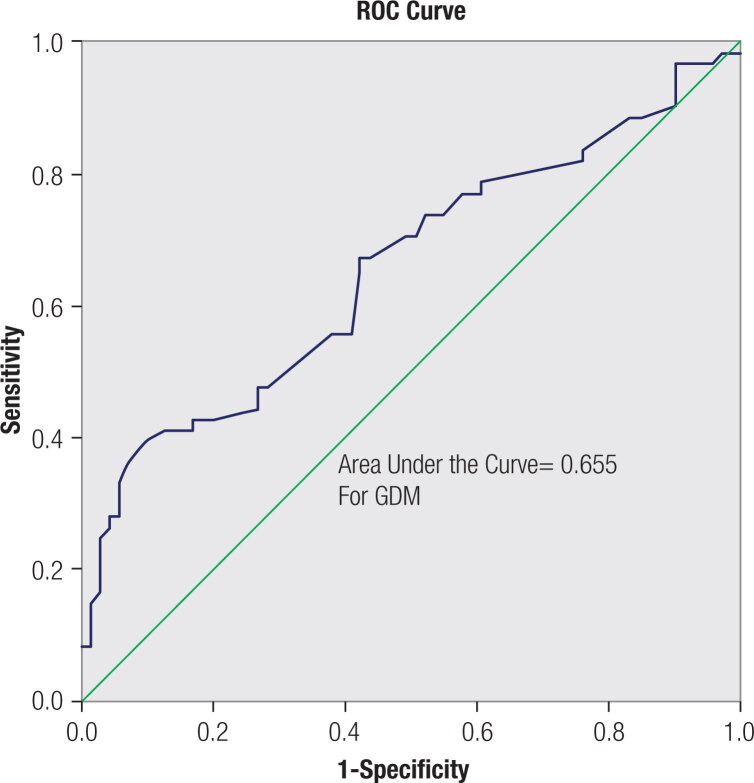
ROC curve of the neck circumference in relation to diagnosis of gestational diabetes mellitus in woman with overweight or obesity.

In the ROC analysis, the area under the curve was 0.655, and the cut-off value of 34.5 cm had a sensitivity of 0.70 and a specificity of 0.51 (Figure 1).

**Figure 2:**
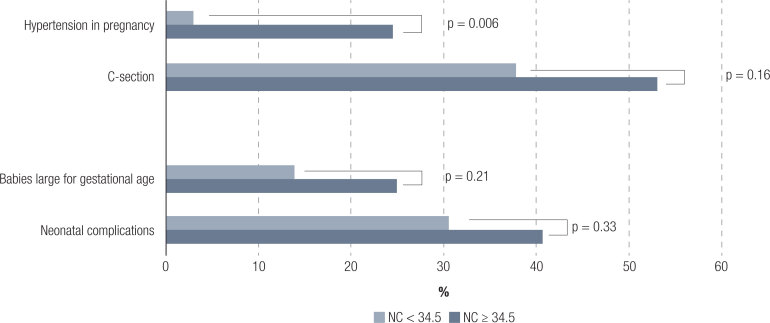
Percentage of maternal-fetal outcomes according to neck circumference (NC) cut off (< or ≥ 34.5 cm).

Women with NC of ≥ 34.5 cm during pregnancy had higher pre-gestational BMI [28.3 (2.9) *vs.* 30.9 (3.9), p < 0.001], systolic blood pressure [109 (11) *vs.* 113 (11), p = 0.027] and diastolic blood pressure [66 (8) *vs.* 71(9), p = 0.002] than those NC < 34.5 cm. Regarding the association between NC and occurrence of maternal-fetal outcomes, women with greater NC cut off had higher rate of hypertension in pregnancy (p = 0.006), and higher but non-significant rate of c-section, infants large for their gestational age, and neonatal complications (Figure 2).

## DISCUSSION

Overweight and obesity are major risk factors for GDM and their prevalence among women of reproductive age is increasing. Identifying risk factors for GDM can help optimize screening strategies and interventions. In the present study, we evaluated the association of NC with GDM diagnosis and maternal-fetal outcomes in women with pre-gestational overweight or obesity. Our results showed that NC was directly associated with plasma glucose level and blood pressure, and with GDM diagnosis. The cut-off of NC for the GDM status was determined using the ROC analysis and settled at 34.5 cm. NC ≥ 34.5 cm was also associated with hypertension in pregnancy for the whole sample.

GDM diagnosis has already been directly associated with some risk factors such as pre-gestational BMI, age, physical activity, education and familiar history of diabetes, but few studies have evaluated NC in pregnancy with GDM (6). Of note, the GDM and non-GDM groups in the actual study had similar BMI, since overweight or obesity (BMI ≥ 25 kg/m^2^) were the eligibility criteria for this study. All women with overweight/obesity are considered to be at equally high risk of GDM, whereas many do not develop the disorder. In this context, a marker of central fat distribution may be relevant considering its prediction role for insulin resistance. Waist circumference is an anthropometric measurement well established as a marker of visceral fat and is a risk factor for diabetes (8), although, not suitable during pregnancy. Neck circumference is another measurement that has also been shown as a marker of central fat accumulation and is associated with the components of metabolic syndrome; It has relevant utility for pregnant women and can be a feasible measurement to be incorporated into clinical practice, including in remote areas. Also relevant was the observation in the actual study that NC had little variation throughout the trimesters, which could represent that this measurement may be accessed at different stages of pregnancy.

Woman with overweight or obesity with GDM had greater neck circumference values than the nonGDM group in the study. Other studies have also show that maternal age, BMI before pregnancy, and maternal NC were significantly higher in then GDM group than in the control group (4,7). In the logistic regression analysis, we observed that NC in pregnancy was independently associated with GDM diagnosis after adjustment for important variables known to be risk factors of hyperglycemia in pregnancy, such as age, physical activity, education and familiar history of diabetes. In this same line, a study considering the confounders in early gestation found that NC and age were independent risk factors of GDM development (6). Another multicenter study in Europe, on > 900 pregnant women with overweight or obesity, found that NC in pregnancy was one of the main predictors for early detection of GDM and overt diabetes (<20 weeks), along with previous abnormal glucose tolerance and previous GDM (9). In the UPBEAT trial, neck/thigh ratio superseded BMI, as an independent variable for GDM at 27-28 weeks (10).

Regarding the importance of age for GDM, we found that women with GDM were older than those without GDM. According to a recent systematic review, age as a risk factor of GDM is an almost universal phenomenon (11). The authors reviewed 24 articles, with a total of 127,275,067 pregnant women. Considering women aged 20-24 years as the reference group, they found that older women showed progressively increase in GDM risk according to age. The ORs for pregnant women aged 25-29, 30-34, 35-39, and ≥ 40 years were 1.69 (95% CI = 1.49-1.93, I 2 97.5%, P < 0.001), 2.73 (95% CI = 2.28-3.27, I 2 98.8%, P < 0.001), 3.54 (95% CI = 2.88-4.34, I 2 98.8%, p < 0.001) and 4.86 (95% CI = 3.78-6.24, I 2 98.6%, p < 0.001), respectively (11). We adjusted our regression models for age and the association of NC with GDM persisted.

Our results also showed that NC, as a continuous variable, was independently and directly associated with systolic and diastolic blood pressure and 2-h plasma glucose from OGTT after adjustment for age and pre-gestational BMI. Our results are similar to some literature data. Hancerliogullari and cols. found an association between neck circumference and plasma glucose after 50 g OGTT (12). Regarding the association with blood pressure, a recent review with 32 studies analyzed, showed that NC had a significant direct correlation with systolic and diastolic blood pressure in adults (13). Our findings of the association between NC and a worse cardiometabolic profile may corroborate the hypothesis that this anthropometric measurement also represents a central fat distribution related to insulin resistance syndrome in pregnancy.

Once the association between NC and GDM and cardiometabolic profile found during pregnancy, it would be interesting to identify a cutoff point that could recognize women at higher risk for GDM. In our study, we found the NC cutoff value of 34.5 cm, with a sensitivity of 0.70 and specificity of 0.51 for the presence of GDM, for women with overweight or obesity. Four studies also evaluated the ideal cutoff of NC in healthy low-risk pregnant women for GDM. Li and cols. (n 371) found a value of 33.8 cm with a sensitivity of 0.68 and specificity of 0.59 (6), while He and cols. (n 255) showed a cutoff of 35.15 cm with a sensitivity of 0.48 and specificity 0.77 (4). KhushBakht and cols. (n 90) presented a similar number of 35.7 cm with a sensitivity of 0.51 and specificity of 0.81 (7), whereas Hancerliogullari and cols. (n 525) found a value of 38.5 cm (12). These differences may be due to the different populations, since the measurement of neck circumference methodology was the same in all of them. Several studies in different populations, including no pregnant women, also investigated the optimal cutoff point of NC for the association with cardiometabolic risk profile and showed different results (5).

As far as we know, no other study has evaluated the cut-off value of NC with maternal-fetal outcomes. We observed higher frequencies of maternal-fetal outcomes (hypertension in pregnancy, c-section, infants large for gestational age and neonatal complications) in women with NC ≥ 34.5 cm during pregnancy, with statistically significant difference only for hypertension in pregnancy. The study sample may not have been large to show statistical significance, but future studies could appropriately address this issue and clarify if NC can be used as a predictive factor for these outcomes.

Our study has some limitations and strengths. The use of only a Brazilian sample limits the generalizability of the study results and external validity, but the results would be important for the Latin and mixed population evaluation. Another limitation when working with anthropometric measurement is interobserver variability. However, in our study, the measurements performed by a single evaluator during the entire data collection, thus reducing interobserver variability. Our sample may have been small to show statistical differences in the frequencies of C-section, large gestational age infants and neonatal outcomes, but we observed a tendency in this direction. On the other hand, the statistical significance for the risk of GDM and hypertension in pregnancy, even with the small sample, emphasizes the importance of NC as a risk factor for these morbidities.

In conclusion, our findings show a direct association of NC during pregnancy with diagnosis of GDM and with hypertension in pregnancy. Our results favor the use of NC, as an easy and feasible assessment during pregnancy, contributing to the prediction of risk of GDM and undesirable maternal-fetal outcomes.
